# Biosurfactants in Plant Protection Against Diseases: Rhamnolipids and Lipopeptides Case Study

**DOI:** 10.3389/fbioe.2020.01014

**Published:** 2020-09-08

**Authors:** Jérôme Crouzet, Anthony Arguelles-Arias, Sandrine Dhondt-Cordelier, Sylvain Cordelier, Jelena Pršić, Gregory Hoff, Florence Mazeyrat-Gourbeyre, Fabienne Baillieul, Christophe Clément, Marc Ongena, Stéphan Dorey

**Affiliations:** ^1^Unité RIBP EA 4707, SFR Condorcet FR CNRS 3417, University of Reims Champagne-Ardenne, Reims, France; ^2^MiPI laboratory, Gembloux Agro-Bio Tech, SFR Condorcet FR CNRS 3417, University of LieÌge, Gembloux, Belgium

**Keywords:** rhamnolipids, lipopeptides, antimicrobial, plant immunity, elicitor, plant pathogen

## Abstract

Biosurfactants are amphiphilic surface-active molecules that are produced by a variety of microorganisms including fungi and bacteria. *Pseudomonas*, *Burkholderia*, and *Bacillus* species are known to secrete rhamnolipids and lipopeptides that are used in a wide range of industrial applications. Recently, these compounds have been studied in a context of plant-microbe interactions. This mini-review describes the direct antimicrobial activities of these compounds against plant pathogens. We also provide the current knowledge on how rhamnolipids and lipopeptides stimulate the plant immune system leading to plant resistance to phytopathogens. Given their low toxicity, high biodegradability and ecological acceptance, we discuss the possible role of these biosurfactants as alternative strategies to reduce or even replace pesticide use in agriculture.

## Introduction

Plant pathogens cause significant agricultural damages ranging from 10 to 40% depending on the crops before or after harvest, resulting in huge economic losses (Savary et al., 2019). Diseases and pests are therefore major problems for sustainable agriculture in the world. Chemical pesticides are largely used to control plant disease worldwide (Popp et al., 2013). However, chemical pesticides can be detrimental to human and environmental health and therefore, development and optimization of alternative strategies to reduce their utilization for crop protection is becoming a necessity. Biocontrol is a promising strategy based on the use of organisms that decrease disease pressure by competing with the pathogen for space and nutrients, by inducing the plant’s natural defense system, and/or by the production of antimicrobial substances (Berg et al., 2017; Bonanomi et al., 2018; Syed Ab Rahman et al., 2018). In addition, natural, ecofriendly and biodegradable compounds isolated from (micro)organisms can also be part of a biocontrol strategy. These compounds can act directly onto the pathogen *via* antimicrobial properties or by stimulating the plant immune system to prime plant protection against disease (Delaunois et al., 2014; Bardin et al., 2015; Keswani et al., 2019). Molecules from microbial origin stimulating the plant immune system are called invasion patterns or elicitors and are highly diverse both in nature and origins (Vatsa et al., 2010; Delaunois et al., 2014; Burketova et al., 2015; Schellenberger et al., 2019).

The main classes of microbial biosurfactants are represented by mannosylerythritol lipids (MEL), trehalose dimycolate (TDM), trehalolipids, sophorolipids, rhamnolipids and lipopeptides. They are used in detergent and cleaning solutions and display a very wide range of applications such as food industry, soil and water remediation, microbial enhanced oil recovery, biomedical science, cosmetic industry, nanotechnology and agriculture (Naughton et al., 2019; Singh et al., 2019). They have been studied since decades in biomedical sciences, especially for their antimicrobial properties and as modulators of human innate immunity [for extensive reviews see (Singh and Cameotra, 2004; Banat et al., 2010; Vatsa et al., 2010; Naughton et al., 2019; Coelho et al., 2020; Jahan et al., 2020; Sajid et al., 2020)]. Some of these microbial biosurfactants exhibit antimicrobial properties that are effective against a large panel of plant pathogens (Mnif and Ghribi, 2016; Penha et al., 2020). In addition, rhamnolipids and lipopeptides have been shown to stimulate the plant immune system conferring a better resistance to fungal and bacterial pathogens (D’aes et al., 2010; Raaijmakers et al., 2010; Vatsa et al., 2010; Schellenberger et al., 2019). In this review, we will provide current knowledge and recent advances on the role of biosurfactants in plant protection. We will focus on rhamnolipids and lipopeptides since these biosurfactants are the most studied for agricultural applications and are among the most effective and economically profitable for industrial production.

## Biosurfactants as Biopesticides

The main interest in the use of biosurfactants as biopesticides in disease management is their environmental-friendly characteristics, high biodegradability and production from renewal energy resources. Rhamnolipids and lipopeptides have been extensively studied in the context of crop protection. Conversely, other biosurfactants such as sophorolipids, MEL and cellobiose lipids have marginally been investigated for their antimicrobial properties toward plant pathogens (Yoshida et al., 2015; Mnif and Ghribi, 2016; Sen et al., 2017; Chen et al., 2020; Penha et al., 2020). Because rhamnolipids and lipopeptides display a good balance between industrial production, efficacy and preservation of the environment, they are very interesting candidates in biocontrol strategies.

### Rhamnolipids Are Efficient Bio-Fungicides

Rhamnolipids are glycolipids produced by various bacterial species including some *Pseudomonas* sp. and *Burkholderia* sp. (Abdel-Mawgoud et al., 2010). Whereas no direct or robust evidences have been reported for rhamnolipid antibacterial or antiviral activities against plant pathogens, a large number of studies described their antifungal activities on pathogens affecting crops. These activities were mainly targeted to fungi and oomycetes including *Botrytis* sp., *Rhizoctonia* sp., *Fusarium* sp., *Alternaria* sp., *Pythium* sp., *Phytophthora* sp., or *Plasmopara* sp. species (Table 1). In these different studies, rhamnolipids, mainly originating from *P. aeruginosa*, were applied either as a mixture or purified molecules. Among all the congeners that are present in the mixtures, purified mono-rhamnolipids (Rha-C_10_-C_10_) and di-rhamnolipids (Rha-Rha-C_10_-C_10_) generally displayed the strongest activity. Moreover, all these studies demonstrated a canonical antimicrobial effect such as zoospore lysis, spore germination abortion and mycelial growth inhibition (Table 1). Because of their amphiphilic nature, glycolipids should be able to interact directly with plasma membranes (Otzen, 2017). It was thus proposed that the mode of action of rhamnolipids against zoospore-producing plant pathogens could be a direct lysis of zoospores *via* the intercalation of the glycolipids within plasma membranes which are not protected by a cell wall (Stanghellini and Miller, 1997). Rhamnolipids could also affect mycelial cells resulting in their destabilization or lysis. Rhamnolipid partition into membranes strongly depends on lipid composition (Aranda et al., 2007). It was shown that purified mono and di-rhamnolipids are able to intercalate into phosphatidylcholine and phosphatidylethanolamine bilayers, notably altering their packing (Ortiz et al., 2006; Sánchez et al., 2006, 2009; Abbasi et al., 2012, 2013). These insertions thus produce structural perturbations, which might affect the function of the membranes. These compounds also alter the physicochemical properties of the bilayer and disturb the hydration status of the water/lipid interface. Depending on the lipid composition of the membrane and on their concentration, rhamnolipids are also able to permeabilize membranes (Sánchez et al., 2010) that could result in their lysis. Amphiphilic compounds such as glycolipids form aggregates in solution depending on their concentration. Above their critical micelle concentration (CMC), they will be present in both forms of aggregates and monomers. Although glycolipid biosurfactants are described to be stable over extreme conditions of pH and temperature (Mnif and Ghribi, 2016), these external factors could also influence rhamnolipid CMC, and therefore their aggregation into micelles (Zhong et al., 2015). The change in CMC could in turn affect their efficiency as biopesticide. Depending on their conformation, the glycolipids could potentially reach more or less easily the pathogen membrane (Aslam et al., 2009) to provoke its disruption (Table 1).

**TABLE 1:** Anti-phytopathogenic properties of rhamnolipids.

Compositions	Source organisms	Sensitive phytopathogens	Effects	References
Rhamnolipids	*Pseudomonas spec. DSM 2874*	*Glomerella cingulata*	Conidial germination inhibition, Growth inhibition (MIC)	Lang et al., 1989
Rha-Rha-C_10_-C_10_, Rha-C_10_-C_10_	*Pseudomonas aeruginosa*	*Phytophthora capsici, Pythium aphanidermatum, Plasmopara lactucae-radicis*	Zoospore lysis	Stanghellini and Miller, 1997
Rha-Rha-C_10_-C_10_	*Pseudomonas aeruginosa* B5	*Cercospora kikuchii, Cladosporium cucumerinum, Colletotrichum orbiculare, Cylindrocarpon destructans, Magnaporthe grisea, Phytophthora capsici*	Zoospore lysis, spore germination and hyphal growth inhibition	Kim et al., 2000
Rha-Rha-C_10_-C_10_, Rha-C_10_-C_10_, Rha-Rha-C_10_-C_12_, Rha-C_10_-C_12_, Rha-C_12:1_-C_10_, Rha-C_12:2_, Rha-C_8:2_	*Pseudomonas aeruginosa* AT10	*Botrytis cinerea, Rhizoctonia solani, Colletotrichum gloeosporioides, Fusarium solani, Penicillium funiculosum*	Growth inhibition (MIC)	Abalos et al., 2001
Rha-Rha-C_8_-C_10_, Rha-C_10_-C_8_/Rha-C_8_-C_10_, Rha-Rha-C_8_-C_12:1_, Rha-Rha-C_10_-C_10_, Rha-Rha-C_10_-C_12:1_, Rha-C_10_-C_10_, Rha-Rha-C_10_-C_12_/Rha-Rha-C_12_-C_10_, Rha-C_10_-C_12:1_/Rha-C_12:1_-C_10_, Rha-Rha-C_12:1_-C_12_, Rha-Rha-C_10_-C_14:1_, Rha-C_10_-C_12_/Rha-C_12_-C_10_	*Pseudomonas aeruginosa* 47T2	*Penicillium funiculosum*, *Fusarium solani*, *Botrytis cinerea*, *Rhizoctonia solani*	Growth inhibition (MIC)	Haba et al., 2003
Rha-Rha-C_10_-C_10_, Rha-C_10_-C_10_, Rha-Rha-C_10_-C_12:1_, Rha-C_10_-C_12:1_, Rha-Rha-C_10_-C_12_, Rha-C_10_-C_12_	*Pseudomonas aeruginosa* LBI	*Penicillium funiculosum, Alternaria alternata*	Growth inhibition (MIC)	Benincasa et al., 2004
Biosurfactant PRO1 (formulation of 25% RLs) Plant support (the Netherlands)	*Pseudomonas aeruginosa*	*Phytophthora cryptogea*	Zoospore lysis, reduction of sporangia formation	De Jonghe et al., 2005
Mono- and di-rhamnolipids	*Pseudomonas aeruginosa* IGB 83	*Phytophthora capsici*, *Phytophthora nicotianae*, *Phytophthora cactorum*, *Phytophthora infestans*, *Pythium aphanidermatum*, *Pythium ultimum*	Motility inhibition, zoospore lysis, mycelial growth inhibition	Yoo et al., 2005
Rha-Rha-C_10_-C_10_, Rha-C_10_-C_10_ (Jeneil Biosurfactant Company JBR599) Biosurfactant PRO1 (formulation of 25% RLs) Plant support (the Netherlands)	*Pseudomonas aeruginosa*	*Pythium myriotylum*	Mycelial growth inhibition	Perneel et al., 2008
Rha-Rha-C_10_-C_10_, Rha-C_10_-C_10_ (Jeneil Biosurfactant Company JBR599)	*Pseudomonas aeruginosa*	*Botrytis cinerea*	Spore germination and mycelial growth inhibition	Varnier et al., 2009; Monnier et al., 2018
Rha-Rha-C_10_-C_10_, Rha-C_10_-C_10_	*Pseudomonas aeruginosa* ZJU211	*Phytophthora infestans, Phytophthora capsici, Botrytis cinerea, Fusarium graminearum*, *Fusarium oxysporum*	Mycelial growth Inhibition	Sha et al., 2012
Rha-C_8:1_, Rha-C_10_-C_10:1_, Rha-C_10:1_-C_10_, Rha-Rha-C_10_-C_12:1_, Rha-Rha-C_12:1_-C_10_	*Pseudomonas aeruginosa* DS9	*Fusarium sacchari*	Mycelial growth Inhibition	Goswami et al., 2014
Mono- and di-rhamnolipids	*Pseudomonas aeruginosa* ZJU-211	*Alternaria alternata*	Spore germination and mycelial growth inhibition	Yan et al., 2014; Yan et al., 2015
Rha-C_10_-C_10_, Rha-Rha-C_10_-C_8_, Other Rha or Rha-Rha: -C_10_-C_10_, -C_8_-C_10_, -C_10_-C_12_, -C_12_-C_12_, -C_14_-C_10_, -C_10_-C_16_	*Serratia rubidaea* SNAU02	*Fusarium oxysporum, Colletotrichum gloeosporioides*	Mycelial growth Inhibition	Nalini and Parthasarathi, 2014
Various mixtures of mono- (Rha) or di-rhamnolipids (Rha-Rha): -C_10_-C_8_/-C_8_-C_10_, -C_10_-C_10:1_/-C_10:1_-C_10_, -C_10_-C_10_, -C_10_-C_12:1_/-C_12:1_-C_10_/-C_10:1_-C_12_/-C_12_-C_10:1_, -C_10_-C_12_/-C_12_-C_10_	*Pseudomonas aeruginosa*	*Phytophthora sojae*	Zoospore motility inhibition	Miao et al., 2015
Rha-Rha-C_8_-C_10_, Rha-C_8_-C_10_, Rha-Rha-C_10_-C_10_, Rha-C_10_-C_10_, Rha-Rha-C_10_-C_12_, Rha-C_10_-C_12_ and purified Rha-Rha-C_10_-C_10_ or Rha-C_10_-C_10_	*Pseudomonas aeruginosa*	*Phytophthora sojae*	Zoospore motility and mycelial growth inhibition	Dashtbozorg et al., 2016
Rha-C_9:2_, Rha-C_10_, Rha-C_12:3_, Rha-C_8_-C_8_, Rha-C_10_-C_10:1_, Rha-C_10:1_-C_10_, Rha-C_10_-C_8_, Rha-C_8_-C_10_, Rha-Rha-C_10_-C_12_, Rha-Rha-C_12_-C_10_	*Pseudomonas aeruginosa* SS14	*Fusarium oxysporum* f. sp. *pisi*	Fungal growth inhibition	Borah et al., 2015
Rha-C_10_-C_10_, Rha-Rha-C_10_-C_10_	*Pseudomonas aeruginosa* KVD-HM52	*Fusarium oxysporum*	Mycelial growth and fungal biomass accumulation inhibition	Deepika et al., 2015
Rha-C_8:2_, Rha-C_8:1_, Rha-C_10_, Rha-C_12:1_, Rha-Rha-C_10:1_, Rha-C_10_-C_10:1_/Rha-C_10:1_-C_10_	*Pseudomonas aeruginosa* DS9	*Colletotrichum falcatum*	Spore germination and mycelial growth inhibition	Goswami et al., 2015
Rha-C_8_, Rha-C_10_-C_10_	*Pseudomonas aeruginosa* SS14	*Fusarium verticillioides*	Spore germination and mycelial growth inhibition	Borah et al., 2016
Rha-Rha-C_10_, Rha-Rha-C_8_-C_10_, Rha-Rha-C_10_-C_10_	*Pseudomonas aeruginosa* DR1	*Sclerotium rolfsii*, *Fusarium oxysporium*, *Phytophthora nicotianae*, *Macrophomina phaseolina*	Mycelial growth inhibition	Sathi Reddy et al., 2016
Rha-Rha-C_10_-C_10_, Rha-C_10_-C_10_	*Pseudomonas aeruginosa* ZJU211	*Verticillium dahliae*	Spore germination and mycelial growth inhibition	Sha and Meng, 2016
Rha-C_10_-C_8_, Rha-C_10_-C_10_, Rha-C_10_-C_12:1_, Rha-C_10_-C_12_, Rha-Rha-C_8_-C_10_, Rha-Rha-C_10_-C_10_, Rha-Rha-C_10_-C_12:1_, Rha-Rha-C_10_-C_12_	Pseudomonas *aeruginosa* #112	*Aspergillus carbonarius*	Mycelial growth inhibition	Rodrigues et al., 2017
Semipurified rhamnolipid mixture (RL90-A, AGAE Technologies, Corvalis, United States) and RL90-N, NatSurFact, Fairfax, United States)	*Pseudomonas aeruginosa*	*Leptosphaeria maculans*	Mycelial growth inhibition	Monnier et al., 2020

### Lipopeptides as Antimicrobial Agents

Lipopeptide biosurfactants (LPs) are composed of a lipid tail linked to a short linear or cyclic oligopeptide. They are produced by fungi and various bacterial genera mainly in the cyclized form and have received considerable attention for their antimicrobial, cytotoxic, antitumor, immunosuppressant and surfactant properties (Raaijmakers et al., 2010). Cyclic lipopeptides (CLPs) represent a class of biosurfactant widely produced by various bacterial species referred as plant-beneficial bacteria (Ongena and Jacques, 2008; D’aes et al., 2010). Among them, *Bacillus* and *Pseudomonas* are exploited as biocontrol agents and are also the best known for the production of a range of structurally distinct and multifunctional CLPs with strong biological activities related to plant protection (D’aes et al., 2010; Raaijmakers et al., 2010). Bacterial CLPs are powerful biosurfactants retaining strong destabilizing activities on biological membranes. Their antimicrobial activity is well documented in a context of biocontrol *via* direct inhibition of phytopathogens. *In vitro*-based assays using purified CLPs combined or not with loss of function mutants of natural producers have highlighted the extremely wide range of fungal and oomycete plant pathogens that are affected by bacterial CLPs such as fengycins and iturins (see recent reviews (Caulier et al., 2019; Rabbee et al., 2019) for *Bacillus* and (Geudens and Martins, 2018; Götze and Stallforth, 2020) for *Pseudomonas* CLPs, respectively). Many studies indicate that CLP activity is linked to their capacity to compromise the fungal cell membrane stability, resulting in cytoplasm leakage and hyphae death or inhibition of spore germination (Chitarra et al., 2003; Romero et al., 2007; Etchegaray et al., 2008; Pérez-García et al., 2011; Gong et al., 2015; Qian et al., 2016). However, the mechanistic basis of antifungal activity may be more complex and, as for rhamnolipids, the lipid composition of the targeted cell membrane could play an essential role in the microbicidal activity (Grau et al., 1999; Tao et al., 2011; Wise et al., 2014). Like other antimicrobial peptides, CLPs are not only membrane disruptive but can also directly or indirectly act on intracellular targets and alter fungal cell functions (Latoud et al., 1987; Qi et al., 2010).

Antibacterial activity has also been occasionally reported for *Bacillus* CLPs such as iturin A, bacillomycin and locillomycins toward several plant pathogens of agronomic importance (Zeriouh et al., 2011; Luo et al., 2015; Cao et al., 2018). However, there are globally few convincing evidences for a direct bactericidal effect of *Bacillus* CLPs and surfactin in particular on phytopathogens or soil-borne bacterial attackers. The precise antibiotic mechanistic of *Bacillus* CLPs against bacterial phytopathogens remains unclear even if a direct interaction with the cellular membrane of the target is also obvious (Zeriouh et al., 2011; Gao et al., 2017). However, in some instances, the inhibitory effect of some *Bacillus* CLPs such as surfactin (or related lichenysin and pumilacidin) is not related to a direct effect on target cell viability but rather due to some interference with key developmental processes of the pathogen such as efficient biofilm formation by *Pseudomonas syringae* and *Ralstonia solanacearum* (Bais et al., 2004; Chen et al., 2013; Xiu et al., 2017) or inhibition of aerial hyphal development of *Streptomyces coelicolor* (Straight et al., 2006; Hoefler et al., 2012).

## Stimulation of Plant Immunity by Biosurfactants

Plants have developed complex defense mechanisms leading to enhance resistance to phytopathogens. After microbial perception, early signaling events are set up including ion fluxes, reactive oxygen species (ROS) accumulation and phosphorylation cascades (Garcia-Brugger et al., 2006; Bigeard et al., 2015). These early signaling and the activation of an intricate network of phytohormones, such as salicylic acid or jasmonic acid, regulate late defense-related responses (Pieterse et al., 2012) including synthesis of antimicrobial metabolites and cell wall reinforcement. These defense responses collectively allow local plant immunity (Boller and Felix, 2009). In addition, microbial perception triggers systemic responses that are effective against a large panel of microorganisms in the whole plant (Fu and Dong, 2013; Pieterse et al., 2014). Activation of the plant immune system involves invasion patterns (IPs) molecules also known as elicitors which can originate from or be produced by the microbe (Schellenberger et al., 2019).

### Rhamnolipids Trigger Local Resistance Against Plant Pathogens

**FIGURE 1:**
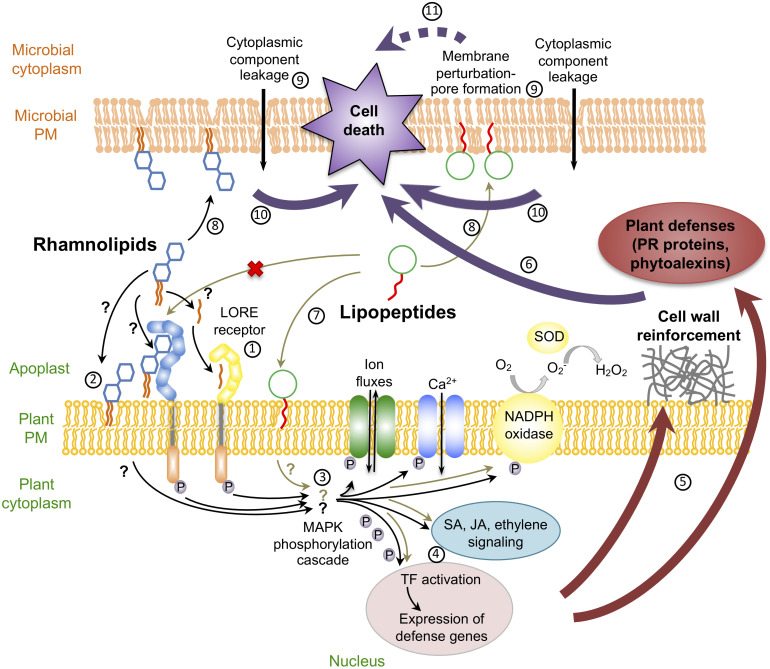
Schematic representation of dual effects of rhamnolipids and lipopeptides: antimicrobial activities and plant defense induction. mc-3-OH-acyl building block of rhamnolipids is perceived by plant through the LORE receptor ①; Rhamnolipid could be sensed through their direct insertion in plasma membrane ②. Recognition of rhamnolipids leads to early signaling events like ion fluxes (Ca^2+^), reactive oxygen species production (H_2_O_2_) and MAPK phosphorylation cascade ③. These early responses trigger defense gene expression, probably through activation of transcription factors (TF) and hormonal signaling pathways ④. This leads to defense mechanisms like cell wall reinforcement and PR protein accumulation ⑤ triggering the resistance to the microbes ⑥. Plant immunity due to lipopeptides does not involve a protein receptor and rely on interaction between lipopeptides and the plant membrane ⑦. Both rhamnolipids and lipopeptides can also have direct antimicrobial effects through direct insertion into the microbial plasma membrane ⑧. These insertions trigger loss of cell morphology leading to pore formation ⑨. The pore formation causes cellular component leakage triggering microbial cell death ⑩. Cell death due to lipopeptides can also be indirectly due to the inhibition or activation of microbial cell functions 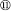.

Whereas most studies on glycolipid biosurfactants were focused on their antimicrobial and antifouling activities, it was recently discovered that rhamnolipids may also stimulate plant innate immunity (Vatsa et al., 2010; Figure 1). Interestingly, despite their antimicrobial and mammalian immunomodulatory properties, to our knowledge, no study on sophorolipids, trehalolipids, MELs or cellobiose lipids, described their potential role in the activation of plant defense responses so far. Following plant sensing, rhamnolipids trigger early signaling events like accumulation of ROS in grapevine and *Brassica napus* (Varnier et al., 2009; Monnier et al., 2018) as well as a calcium influx and a phosphorylation cascade in grapevine (Varnier et al., 2009). Callose deposition, hormone production, defense gene activation and a hypersensitive reaction-like response are also hallmarks of rhamnolipid-triggered immunity in *Brassicaceae* and grapevine (Varnier et al., 2009; Sanchez et al., 2012; Monnier et al., 2018, 2020). It was demonstrated in Arabidopsis that rhamnolipid-mediated local resistance to *Botrytis cinerea, Hyaloperonospora arabidopsidis* or *P. syringae* pv. *tomato* (*Pst*) involves different signaling pathways that depend on the type of pathogen (Sanchez et al., 2012). In addition, rhamnolipid potentiate defense responses induced by other elicitors like chitosan. The immune response triggered by rhamnolipids also participates in local resistance against *B. cinerea* and the hemibiotrophic fungus *Leptosphaeria maculans* in *B. napus* (Monnier et al., 2018, 2020). A large range of rhamnolipid concentrations from 0.005 to 1 mg/mL have been used to induce immunity on these various plant species (Varnier et al., 2009; Sanchez et al., 2012; Monnier et al., 2018, 2020). Synthetic biosurfactants derived from rhamnolipid structure are also elicitors. For instance, synthetic rhamnolipid bolaforms, composed of two rhamnoses separated by a fatty acid chain, trigger an immune response in Arabidopsis that varies according to fatty acid chain length (Luzuriaga-Loaiza et al., 2018). In addition, RL harboring carboxylic acid (Ac-RL) and methyl (Alk-RL) induce ROS production in this plant (Nasir et al., 2017).

The way by which rhamnolipids are perceived by plant cells still remains unknown. Given their amphiphilic nature, it is postulated that they could interact with plant membrane lipids (Sanchez et al., 2012; Schellenberger et al., 2019). Recently it has been demonstrated that natural rhamnolipids fit into plant lipid-based membrane models and are located near the lipid phosphate group of the phospholipid bilayers, nearby phospholipid glycerol backbones (Monnier et al., 2019). Rhamnolipid insertion inside the lipid bilayer does not strongly affect lipid dynamics but the nature of the phytosterols could influence the effect of the glycolipids on plant plasma membrane destabilization. These subtle changes in lipid dynamics could be linked with plant defense induction (Monnier et al., 2019). Interestingly, whereas no receptor for rhamnolipid perception has been identified so far, the mc-3-OH-acyl building block of rhamnolipids is sensed by the lectin S-domain-1 receptor-like kinase LORE (Kutschera et al., 2019; Figure 1).

For some studies, it is not demonstrated whether rhamnolipid-triggered protection is driven by activation of plant defense responses and/or antimicrobial properties. For instance, treatments of pepper plants with rhamnolipids result in an enhanced protection to *Phytophthora* blight disease and also prevent the development of *Colletotrichum orbiculare* infection on leaves of cucumber plants (Kim et al., 2000). Rhamnolipids significantly decrease the incidence of water-borne damping-off disease by *Phytophthora* sp. and *Pythium* sp. (Yoo et al., 2005). Similar results were obtained in field trials on chili pepper and tomato (Sharma et al., 2007a, b). Using bacterial mutants, it was demonstrated that phenazine and rhamnolipids interact in the biological control of soil-borne diseases caused by *Pythium* sp. (Perneel et al., 2008). Syringomycin E and rhamnolipids can also act synergistically to control pathogenic and opportunistic fungi recovered from diseased grape (Takemoto et al., 2010). The control of postharvest phytopathogens on seeds or fruits for a better conservation is often related to antimicrobial activities. But we cannot exclude that protection could also be due to plant defense responses (Borah et al., 2016). When applied alone, rhamnolipids induce antioxidative reactions in cherry tomato fruit, leading to a significant reduction of fungal disease (Yan et al., 2015). When applied in combination with the biocontrol yeast agent *Rhodotorula glutinis*, a synergistic inhibitory effect on *Alternaria alternata* infection could be observed in cherry tomato fruit, leading to an efficient protection (Yan et al., 2014). This protection is associated with a higher induction of defense-related enzymes and the accumulation of antimicrobial metabolites.

### Lipopeptides as Powerful Inducers of Plant Systemic Resistance

Several studies have reported the involvement of *Bacillus* CLPs in plant immunity induction on various pathosystems. The potential of fengycin and surfactin CLPs to trigger plant systemic resistance was first shown on bean and tomato plants. When applied as pure compounds at micromolar concentrations, surfactin and to a lower extend fengycin induced significant disease reduction in bean and tomato infected with *B. cinerea* (Ongena et al., 2007). More recently, a study performed with a large range of natural *Bacillus* isolates strengthened the role of surfactin as ISR (induced systemic resistance) inducer since strong correlation was observed between defense-inducing activity and the amount of surfactin produced by the different strains (Cawoy et al., 2014). In the same way, *B. velezensis* FZB42 mutant strains unable to synthesize surfactin are impaired in their ISR to *Rhizoctonia solani* in lettuce (Chowdhury et al., 2015). Further studies allowed enlarging the ISR elicitor role of surfactin to other plants. For example, purified surfactin was shown to increase resistance against the cucurbit powdery mildew in melon plants (García-Gutiérrez et al., 2013). In the pathosystem citrus fruit/*Penicillium digitatum*, surfactin stimulates defense responses involved in generating signal molecules for ISR activation (Waewthongrak et al., 2014). This lipopeptide activates a plant innate response effective against *Magnaporthe oryzae* in perennial ryegrass (Rahman et al., 2015) or *Plasmopara viticola* in grapevine (Li et al., 2019). It also reduces infection by the rhizomania disease vector *Polymyxa betae* in sugar beet (Desoignies et al., 2013) or by *Colletotrichum gloeosporioides* in strawberry leaves (Yamamoto et al., 2015). Finally, a recent study showed that *Sclerotium rolfsii* disease incidence was strongly reduced in *Arachis hypogaea* when pretreated with surfactin (Rodríguez et al., 2018). Interestingly, CLPs like surfactin do not globally provoke a strong plant defensive response associated with major genetic reprograming and fitness cost but rather act by priming host defenses to trigger systemic resistance (Ongena et al., 2007; Jourdan et al., 2009; Debois et al., 2015). Induction of plant defenses by CLPs of the iturin group has also been occasionally reported. Iturin A was shown to have a similar role as surfactin in strawberry leaves (Yamamoto et al., 2015) and also acted as an inducer of plant defense gene expression in cotton plants upon *Verticillium dahliae* attack (Han et al., 2015). Mycosubtilin is the most efficient lipopeptide inducing an immune response in grapevine (Farace et al., 2015). Compared to surfactin, bacillomycin D produced by *B. velezensis* SQR9 has a comparable efficacy in *Arabidopsis* ISR elicitation to prevent infection by *P. syringae* or *B. cinerea* (Wu et al., 2018). In wheat plants, resistance toward *Zymoseptoria tritici*, was activated by pure surfactin used at concentrations ranging from 1 to 100 μM upon foliar application (Le Mire et al., 2018). Some CLPs synthesized by *Pseudomonas* sp. also display consistent ISR-triggering activity. It was first demonstrated that massetolide A produced by *Pseudomonas fluorescens* strain SS101 retains ISR-eliciting activity in tomato plants for the control of *Phytophthora infestans* (Tran et al., 2007). *Pseudomonas* sp. strain CMR12a is a soil isolate retaining high biocontrol potential against *R. solani* relying mainly on the interplay between two different lipopeptides (sessilin and orfamide) and phenazine for inducing plant immunity (D’aes et al., 2011, 2014). These CLPs were also active at protecting *Brassica chinensis* against *R. solani* (Olorunleke et al., 2015). In monocots, such as rice, orfamide and other *Pseudomonas* CLPs such as WLIP, lokisin and entolysin, successfully induced resistance toward *C. miyabeanus* or *M. oryzae* (Ma et al., 2016, 2017; Omoboye et al., 2019).

Up to now, how lipopeptides act and are recognized by plant cells to activate ISR remains unclear. CLPs are in most instances only active in micromolar concentrations, and defenses are more intensively induced at the highest surfactin doses. This suggests that the recognition mechanism at the plant cell surface should be of quite low affinity in contrast to other elicitors (Jourdan et al., 2009). Such a low specificity may be explained by the fact that CLPs like surfactin are not perceived by a protein receptor, but rather involve a process driven by an uncommon pathway based on interaction with the lipid bilayer fraction of plant plasma membranes (Henry et al., 2011). This is supported by some studies revealing that CLP structure plays an important role for the ISR eliciting activity. Both the fatty acid chain length, the cyclic conformation of the molecule and amino acid positions in the peptide chain impact the eliciting potential of surfactin in tobacco cells (Jourdan et al., 2009; Henry et al., 2011). The activation of defense genes in Arabidopsis upon iturin A foliar treatment was also dependent on the structure of the molecule, i.e., cyclization and/or nature of the fatty acid chain (Kawagoe et al., 2015). As for rhamnolipids, the hypothesis is that lipopeptides have the ability to create some disturbance in the plant plasma membrane and could consequently activate a cascade of molecular events leading to the activation of defense mechanisms (Schellenberger et al., 2019; Figure 1).

## Conclusion

Biosurfactants, produced by bacteria, yeast, and fungi, are promising molecules for a wide variety of applications due to their potential to be commercially produced at large scales, their low toxicity and high biodegradability. In this mini-review, we provided evidences about the potential of rhamnolipids and lipopeptides for plant protection in a context of sustained agriculture. These molecules have similar dual effects by protecting plants through antimicrobial properties and stimulation of local and/or systemic plant immunity. These singular properties are essential for the efficiency of these biopesticides. Although numerous elicitors are perceived by plasma membrane receptors, recent studies on amphiphilic biosurfactants such as rhamnolipids or lipopeptides suggest that they are sensed by an uncommon way involving lipids in the bilayer of the plant plasma membrane that could explain their singular elicitor activity. To better understand the mechanisms of action of biosurfactants, experiments or trials need to be realized not only on mixture but also on highly purified molecules in the future. Nevertheless, several obstacles to the development of rhamnolipid and lipopeptide applications still remain. Biosurfactant costs, their efficacies in the field and purity of compounds have to be improved to allow their use at a higher degree in crop protection. In this respect, combination of biosurfactants should be considered to increase efficacy in field conditions. Finally, given their interesting properties it is now time to really consider ecofriendly biosurfactants as biocontrol solutions in integrated pest management.

## Author Contributions

JC, AA-A, SD-C, SC, MO, and SD participated in the conception, information search and assisted in drafting the manuscript. JP, GH, FM-G, FB, and CC assisted in drafting and correction of the manuscript. All authors contributed to the article and approved the submitted version.

## Conflict of Interest

The authors declare that the research was conducted in the absence of any commercial or financial relationships that could be construed as a potential conflict of interest.
